# *Trichoderma*-Inoculation and Mowing Synergistically Altered Soil Available Nutrients, Rhizosphere Chemical Compounds and Soil Microbial Community, Potentially Driving Alfalfa Growth

**DOI:** 10.3389/fmicb.2018.03241

**Published:** 2019-01-07

**Authors:** Fengge Zhang, Xixi Xu, Yunqian Huo, Yan Xiao

**Affiliations:** College of Agro-Grassland Science, Nanjing Agricultural University, Nanjing, China

**Keywords:** high-throughput sequencing, rhizosphere soil chemical compounds, soil microbial community, Pearson’s correlation, structure equation modeling

## Abstract

*Trichoderma* spp. are proposed as major plant growth-promoting fungi (PGPF) to increase plants growth and productivity. Mowing can stimulate aboveground regrowth to improve plant biomass and nutritional quality. However, the synergistic effects of *Trichoderma* and mowing on plants growth, particularly the underlying microbial mechanisms mediated by rhizosphere soil chemical compounds, have rarely been reported. In the present study, we employed *Trichoderma harzianum* T-63 and conducted a pot experiment to investigate the synergistic effect of *Trichoderma*-inoculation and mowing on alfalfa growth, and the potential soil microbial ecological mechanisms were also explored. Alfalfa treated with *Trichoderma-*inoculation and/or mowing (T, M, and TM) had significant (*P* < 0.05) increases in plant shoot and root dry weights and soil available nutrients (N, P, and K), compared with those of the control (CK). Non-metric multidimensional scaling (NMDS) demonstrated that the rhizosphere chemical compounds and soil bacterial and fungal communities were, respectively, separated according to different treatments. There was a clear significant (*P* < 0.05) positive correlation between alfalfa biomass and the relative abundance of *Trichoderma* (*R^2^* = 0.3451, *P* = 0.045). However, *Pseudomonas, Flavobacterium, Arthrobacter, Bacillus, Agrobacterium*, and *Actinoplanes* were not significantly correlated with alfalfa biomass. According to structure equation modeling (SEM), *Trichoderma* abundance and available P served as primary contributors to alfalfa growth promotion. Additionally, *Trichoderma*-inoculation and mowing altered rhizosphere soil chemical compounds to drive the soil microbial community, indirectly influencing alfalfa growth. Our research provides a basis for promoting alfalfa growth from a soil microbial ecology perspective and may provide a scientific foundation for guiding the farming of alfalfa.

## Introduction

*Trichoderma* species are important plant growth-promoting fungi (PGPF) in soils that have been reported to significantly facilitate plant growth and development (Singh et al., 2015) through numerous mechanisms: increasing solubilization of soil nutrients (Yadav et al., 2009; Kapri and Tewari, 2010), increasing nutrient efficacy and recycling (Azarmi et al., 2011), releasing plant growth stimulatory agents (Contreras-Cornejo et al., 2009) and inducing systemic resistance (Shoresh et al., 2010). Several studies show that *Trichoderma*-inoculation has significant promotion effects on the growth of many plant species (Howell et al., 2000; Zhang et al., 2013a), including those of pasture fields (Lee et al., 2015; Zhang et al., 2018b). Yuan et al. (2018) has demonstrated that five *Trichoderma* strains (3 strains of *Trichoderma harzianum*, 1 strain of *Trichoderma longibrachiatum* and *Trichoderma reesei*) substantially facilitate the growth and improve the nutritional quality of orchard grass. Zhang et al. (2018a) also showed that bio-fertilizer could effectively manipulate soil microbial communities, and *Trichoderma* was a major contributor to improve *Leymus chinensis* biomass.

Alfalfa (*Medicago sativa* L.) is the most familiar and widely distributed legume in the world, which is characterized by high yields and nutritive value, palatability and digestibility, and plays a particularly important role in animal husbandry development (Radović et al., 2009). Mowing is a common and beneficial management technique that is primarily employed in alfalfa production to store hay, silage or various dehydrated products (Barać et al., 2012). Additionally, the rapid recovery ability of alfalfa after mowing and its tolerance to environmental stress are also important features, which support and maintain stable yield. Mowing is beneficial to plant regrowth by removing the aboveground biomass, with the new leaves then improving the total biomass and plant nutritional quality (Robson et al., 2007; Jensen et al., 2012). Radović et al. (2009) revealed that the first flower (10% blooms) stage is suitable for mowing to obtain the optimum nutrients and yield of alfalfa. However, the synergistic effects of *Trichoderma* and mowing on alfalfa growth, particularly the underlying microbial mechanisms that are mediated by rhizosphere chemical compounds, have rarely been studied.

Rhizosphere soil chemical compounds mainly include plant root exudates, breakdown products and microbial metabolites (Pierre et al., 2017). The rhizosphere chemical compounds participate in the communication between plant roots and their associated microorganisms (Oburger and Schmidt, 2016). Plant roots release a series of compounds that are involved in attracting various organisms and forming associations in the rhizosphere. The review of Dessaux et al. (2016) reported that the soil was altered into complex environments by plant roots to support various microbial communities. Accumulated evidence shows that carbon composition in root exudates largely affects soil bacterial and fungal community composition (Salles et al., 2009; Ng et al., 2014). Soil microorganisms that are present in the rhizosphere microbiota can have profound effects on plant growth, nutrition and health in agro-ecosystems (Bonfante and Anca, 2009; Mendes et al., 2011; Berendsen et al., 2012). High soil microbial diversity and appropriate community composition are significant factors in supporting ecosystem productivity (Bell et al., 2005; Chaer et al., 2009). Philippot et al. (2013) also indicated that a suitable soil microflora is one of the most important elements to promote plant growth.

In this study, we explored the synergistic influences of *Trichoderma*-inoculation and mowing on alfalfa growth and the underlying soil microbial ecological mechanisms. We hypothesized the following: (1) *Trichoderma*-inoculation and mowing amendment would result in an additive effect on the growth of alfalfa; (2) *Trichoderma*-inoculation and mowing would significantly alter the rhizosphere chemical compounds; and (3) Rhizosphere chemical compounds would strongly drive the variations in soil microbial communities, correlating with alfalfa growth.

## Materials and Methods

### *Trichoderma* Strain and Preparation of Conidia Suspension

The preisolated strain, *Trichoderma harzianum* T-63 (CCTCC No. AF2018013, China Center for Type Culture Collection) was used throughout our study, which was provided by the Grassland Microecology and Vegetation Restoration Laboratory, Nanjing Agriculture University, Nanjing, China. Supplementary Figure S1 shows the identification of T-63, which was based on internal transcribed spacer (ITS) sequence analysis. *Trichoderma. harzianum* T-63 was stored at -80°C in potato dextrose agar (PDA) + 30% glycerol and cultured on PDA at 28°C. The preparation of *T. harzianum* T-63 conidia suspension was according to Zhang et al. (2013b), and the determined concentration was 2.9 × 10^8^ colony-forming units (CFU) ml^-1^.

### Greenhouse Experiment

#### Seeds and Soil for Experiment

Alfalfa seeds (*Medicago sativa* L. cv Zhongmu-No. 1) with 98% seed germination rate were used throughout this experiment. The seeds were surface-sterilized according to Hoyos-Carvajal et al. (2009), and then all seeds were removed to plates containing sterile, wet filter paper for germination at 28°C. The soil used in the pot experiment was a healthy soil that collected from Nanjing, Jiangsu Province, which contained 11.6 g kg^-1^ organic matter, 6.73 g kg^-1^ organic C, 0.65 g kg^-1^ total N, 28.4 mg kg^-1^ available P, 62 mg kg^-1^ available K, and pH = 6.5. The soil was mixed with 2.0% organic fertilizer (35.4% organic matter, 3.0% N, 2.2% P_2_O_5_, and 1.8% K_2_O) for the following pot experiment.

#### Experimental Design

Pre-germinated alfalfa seeds were transferred in each plastic pot (3 kg soil), and three seedlings remained after sowing. Four treatments were established in this experiment: (1) Control (no *Trichoderma*-inoculation and not mowed); (2) T (*Trichoderma*-inoculation and not mowed); (3) M (mowed and no *Trichoderma*-inoculation); and (4) TM (*Trichoderma*-inoculation and mowed). Each treatment had three blocks, and each block contained 10 plastic pots. All the plastic pots were laid out randomly.

Plants were inoculated with *Trichoderma* for two times at a rate of 100 ml/pot (July 20, 2016 and September 20, 2016). The pots were mowed on September 20, 2016 and October 20, 2016, and vegetation was cut to 10 cm above the soil surface and removed from the pots. All alfalfa plants were grown in a greenhouse with temperatures from 30 to 32°C (day)/24 to 26°C (night), relative air humidity from 60 to 70%, and a photoperiod of 16 h light and 8 h dark, which was located in Nanjing, Jiangsu Province, China; the study was from July to October 2016. The soil was regularly adjusted to maintain 60% water-holding capacity with deionized water during the whole experimental periods to guarantee the growth of alfalfa seedlings.

#### Soil and Vegetation Samples Collection

Soil and vegetation samples were collected from the four treatments on October 20, 2016. A composite rhizosphere soil sample was collected from the roots of all alfalfa plants of a block; thus, each treatment had three composite samples. All fresh soil samples were sieved through a 2.0-mm mesh and homogenized. Each sample was divided into three subsamples: the first subsample was stored at 4°C for soil chemical compounds analysis; the second subsample was air dried at room temperature for soil property analysis; and the third subsample was stored at -20°C for DNA extraction. At harvest, the shoots were cut and then oven-dried at 65°C for 72 h before weighing. For the treatments with mowing, the aboveground biomass was estimated by adding the biomass removed during mowing to the final harvest. The aboveground biomass in unmowed treatment was assessed at the end of harvest. The shoot dry weight is expressed as total aboveground biomass per plant.

### Extraction and Identification of Rhizosphere Soil Chemical Compounds

For each rhizosphere soil sample, the extraction of soil chemical compounds was according to the procedures of Zhang et al. (2018a). In brief, 5.0 g soil and 50 ml of ethyl acetate (1:10 ratio) were placed in a 150 ml Erlenmeyer flask and shaken on a table agitator at 30°C for 2 h. After that, the extracted suspension was filtered (0.45-μm) and concentrated to 500 μl at 35°C using a vacuum rotary evaporator (Yarong Model RE-52A, Shanghai, China). The concentrated solution was analyzed by gas chromatography-mass spectrometry (GC-MS) with the triple quadruple mass spectrophotometer fused silica capillary column BR-5MS 30 m × 0.25 mm × 0.25 μm (Bruker, Germany). The mass spectrometry detector (MSD) was operated in electron ionization mode at 70 eV, with a source temperature of 230°C. The initial oven temperature of 60°C was held for 3 min, increased at a rate of 5°C min^-1^ to 240°C, held at 240°C for 5 min, further increased a rate of 20°C min^-1^ to 280°C, and held for 2 additional minutes. A continuous scan from 45 to 500 m/z was applied. Helium was the carrier gas at a linear velocity of 1.0 ml min^-1^. The chromatographic peaks of the components were compared to entries in the National Institute of Standards and Technology (NIST) database (Version 2.0) to characterize the variation in the chemical composition of each soil sample.

### Soil pH and Chemical Properties Measurements

Soil pH was determined with soil and water with the ratio of 1:5 (w/v) using a compound electrode (PB-10; Sartorius, Germany). Soil organic C (SOC) and soil total N (TN) were determined with an Elementar Analyzer (Vario EL III, Germany). Soil available N was measured according to Shi (1996). Soil total P was determined according to Olsen and Sommers (1982), and soil available phosphorus in the soil (Olsen-P) was measured using the molybdenum-blue method (Watanabe and Olsen, 1965). Soil total and available K were extracted with ammonium acetate and measured by flame photometry (Knudsen et al., 1982).

### Rhizosphere Soil DNA Extraction

For each rhizosphere soil sample, the total genomic DNA was extracted from 0.5 g of soil with a Power Soil DNA Isolation Kit (MoBio Laboratories Inc., Carlsbad, CA, United States) according to the manufacturer’s instructions. DNA was eluted with 75 μl of elution solution from the kit. The DNA sample concentration and quality (A260/A280 ratio) were measured using a NanoDrop 2000 spectrophotometer (Thermo Scientific, Waltham, MA, United States). Each treatment had three replicates in our experiment.

### Quantitative Analysis of *Trichoderma* With TaqMan Real-Time PCR

The primer pair (ITS1 S: 5′-TACAACTCCCAAACCCAATGT GA-3; ITS1 R: 5′-CCGTTGTTGAAAGTTTTGATTCATTT-3′) (Lopez-Mondejar et al., 2010) and probe 5′-FAM-AACTCTTTTTGTATACCCCCTCGCGGGT-TMR-3′ (FAM: 6-carboxyfluorescein, TAMRA: 6-carboxy-tetramethylrhodamine) (Zhang et al., 2018b) were used to amplify the *Trichoderma* ITS region. In the real-time PCR amplifications, Premix Ex Taq^TM^ (Takara) had a volume of 20 μl: 10 μl of Premix Ex Taq^TM^ (Takara Bio Inc., Japan), 0.4 μl of Rox Reference Dye II (50 ×), 0.4 μl of each primer (10 μM), 0.8 μl of TaqMan probe (10 pM), 2 μl of template DNA and 6 μl of sterilized double-distilled water. The standard curve of *Trichoderma* was generated according to Lopez-Mondejar et al. (2010). The PCR was performed on a 7500 Fast Real-Time PCR System (Applied Biosystems, Foster City, CA, United States) with the following program: 95°C for 30 s, 40 amplification cycles of 5 s at 95°C and at 60°C for 45 s. All the quantification results were analyzed with the 7500 system SDS Software version 1.4 (Applied Biosystems), and the results are expressed as log copy numbers per gram of dry soil.

### High-Throughput Sequencing and Bioinformatics Analysis

High-throughput sequencing analysis of the 16S rRNA and ITS region was performed to determine soil bacterial and fungal communities, respectively. Each treatment had three replicates sequencing. The universal primers 520F (5′-AYT GGG YDT AAA GNG-3′) and 802R (5′-TAC NVG GGT ATC TAA TCC-3′) were employed to amplify the V4 region of the bacterial 16S rRNA gene, and the primers ITS1F (5′-CTT GGT CAT TTA GAG GAA GTA A-3′) and ITS2 (5′-GCT GCG TTC TTC ATC GAT GC-3) were used to amplify the ITS1 region of the fungal ITS. PCR was performed in a 30 μl reaction system: 15 μl 2 × Master Mix (Thermo 193 Scientific^®^Phusion High-Fidelity PCR Master Mix, New England Biolabs, United Kingdom), 0.5 μl of each primer, 10 ng template DNA and nuclease-free water up to 30 μl. The PCR conditions were 95°C for 5 min, 27 cycles of 30 s at 95°C, 30 s at 50°C for 16S rRNA or 61°C for ITS, and 45 s at 72°C, with a final extension of 5 min at 72°C. After PCR amplification, the obtained products were purified using a PCR Purification Kit (Axygen Bio, United States) and quantified with PicoGreen^®^dsDNA reagent (Promega, United States). Then, the purified amplicons were then pooled in equimolar concentrations as a single aliquot and employed for library construction using the NEB Next^®^Ultra^TM^ DNA Library Prep Kit for Illumina (New England Biolabs, United Kingdom). The final quality and concentration of the libraries were checked using Agilent 2100 Bioanalyzer Instruments (Agilent Technologies Co. Ltd., United States) and determined using KAPA Library Quantification Kits (Kapa Biosystems, United States). All of the preparation of the libraries for sequencing was performed on the Illumina MiSeq sequencer at Newgenes Biotechnology Co., Ltd. (Shanghai, China). The raw 16S rRNA and ITS pyrosequencing data were processed using a Quantitative Insights Into Microbial Ecology (QIIME) tool kit (Caporaso et al., 2011) and UPARSE pipeline (Edgar, 2013) according to Shen et al. (2015). The MOTHUR (version 1.25.1) standard operating procedure (SOP) was employed to further analyze the pyrosequencing data (Wang et al., 2007; Schloss et al., 2009).

### Statistical Analyses

The data in our study were log-transformed when necessary to satisfy the criteria for a normal distribution and homogeneity of variance. The differences of plant biomass, soil pH, chemical properties and soil chemical compounds were determined by one way analysis of variance (ANOVA) in IBM SPSS 20 (IBM Corporation, New York, United States) and Duncan’s multiple range tests were performed for multiple comparisons. Moreover, the differences are statistically significant at 0.05 probability level in this study. Non-metric multidimensional scaling (NMDS) plots and principal component analysis (PCA) were both performed in R with the vegan package (Version 3.0.2, Oksanen et al., 2013). SEM was employed to test the direct and indirect pathways among *Trichoderma* abundance, soil available N, P, and K, soil chemical compounds, soil bacterial and fungal communities, and alfalfa biomass. The figures were generated with Sigmaplot 12.0 (Systat Software Inc., CA, United States).

### Accession Number

The obtained bacterial 16S and fungal ITS sequences data are available at the National Center for Biotechnology Information (NCBI) Sequence Read Archive (SRA) database under accession numbers SRP151105.

## Results

### Evaluation of Alfalfa Growth and Soil Properties in Pot Experiment

**FIGURE 1:**
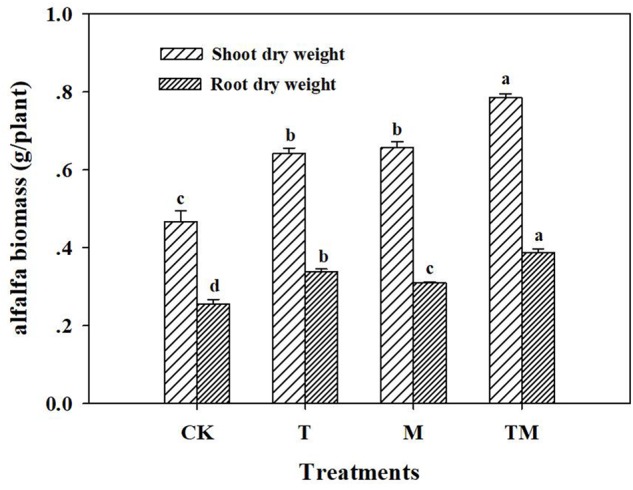
Alfalfa biomass in different treatments. CK: no *Trichoderma*-inoculation and not mowed; T: *Trichoderma*-inoculation and not mowed; M: mowed and no *Trichoderma*-inoculation; TM: mowed and *Trichoderma*-inoculated. Bars above the histogram represent standard deviations and different letters indicate significant differences according to ANOVA test.

Alfalfa treated with *Trichoderma* and/or mowing (T, M, and TM) had significant increases in plant shoot and root dry weights, compared with those of the control (CK). Alfalfa inoculated with *Trichoderma* and mowing (TM) had the highest dry weight (1.17 g/plant) among all treatments (Figure 1). Specifically, TM, M and T treatments significantly increased alfalfa shoot dry weight by 68.1, 22.4, and 19.5%, respectively, compared with the CK. However, no significant difference was detected in shoot dry weight between T and M treatments. The root dry weight in the TM treatment was significantly higher than that in other treatments (M, T, and CK). Moreover, the root dry weight in the *Trichoderma-*inoculation treatment (T) was increased significantly by 9.1%, compared with that of the mowing treatment (M).

As shown in Table 1, no significant difference was observed in pH, organic matter and soil total nutrients (N, P, and K), while the available nutrients (available N, P, and K) were significantly different among the treatments. Compared with the control (CK), *Trichoderma* and/or mowing (T, M, and TM) significantly increased soil available nutrients (N, P, and K). Additionally, the contents of available N, P, and K in TM were significantly higher than those in other treatments (M, T, and CK).

**Table 1:** Soil pH and chemical properties in different treatments.

Soil properties	Treatments			
	CK	T	M	TM
pH	8.0 ± 0.20a	8.0 ± 0.17a	8.1 ± 0.21a	8.0 ± 0.06a
Organic matter (g/kg)	22.3 ± 0.50a	23.1 ± 0.72a	23.3 ± 0.35a	22.7 ± 1.00a
Total N (g/kg)	1.6 ± 0.00a	1.6 ± 0.06a	1.6 ± 0.06a	1.6 ± 0.09a
Total P (g/kg)	0.7 ± 0.06a	0.8 ± 0.06a	0.7 ± 0.00a	0.8 ± 0.06a
Total K (g/kg)	17.4 ± 0.76a	18.1 ± 0.44a	17.4 ± 0.21a	18.1 ± 0.55a
Available N (mg/kg)	90.4 ± 6.13c	118.8 ± 3.80b	110.8 ± 6.31b	176.9 ± 12.15a
Available P (mg/kg)	14.5 ± 0.53c	17.7 ± 0.90b	18.8 ± 0.36b	20.4 ± 0.90a
Available K (mg/kg)	97.3 ± 5.03b	103.1 ± 4.08ab	97.9 ± 5.63b	111.5 ± 10.12a

*CK: no *Trichoderma*-inoculation and not mowed; T: *Trichoderma*-inoculation and not mowed; M: mowed and no *Trichoderma*-inoculation; TM: mowed and *Trichoderma*-inoculated. Data are mean values of three replicates. Numbers followed by “±” are the standard deviations (SDs). Within a row, values that do not share a letter are significantly different (*P* < 0.05) according to Duncan’s test.*

### Variation in Rhizosphere Soil Chemical Compounds

The GC-MS analysis identified 99 compounds, and 59 chemicals were shared by the four treatments (Supplementary Table S1). The identified chemical compounds with relative abundance of that was significantly different among the four treatments are shown in Table 2 and including 17 alkanes, 1 arene, 3 alcohols, 5 esters, 1 acid, 1 amine, and 1 phenol. No Tetratetracontane, Hexadecane,2,6,10,14-tetramethyl-, 1-Hexacosene, (E,E,E,E)-Squalene, Naphthalene,1,2,3,4-tetrahydro-1,8-dimethyl, Dibutyl phthalate, 2,2′-Methylenebis, Oleamide or 2,4-bis(1-phenylethyl)phenol was identified in the CK. Compared with the control (CK), *Trichoderma* and/or mowing (T, M, and TM) significantly increased the relative abundances of beta-Sitosterol and 2,2′-Methylenebis. The relative abundances of 1-Hexacosene and 1-Heptadecanol were significantly higher in the rhizodeposits of TM than in those of other treatments.

**Table 2:** Retention time and peak areas (%) of identified soil chemical compounds, indicating relative abundances across treatments.

Category	ID	Retention time	Kovats index	Name	Peak areas (%)			
					CK	T	M	TM
**Alkanes**	GC4	12.069	1140.74	Hexadecane	0.159a	0.110ab	0.005b	0.196a
	GC8	12.872	1167.82	Undecane, 2,6-dimethyl	0.577ab	0.499ab	0.337b	0.687a
	GC9	13.343	1183.71	9-Methyl heptadecane	0.711a	0.659a	0.110b	0.588ab
	GC11	14.593	1226.59	2,6,10-Trimethyl decane	1.021a	0.422b	0.143b	1.116a
	GC13	14.997	1240.59	N-Hentriacontane	0.040b	0.235ab	0.370a	0.000b
	GC17	15.88	1271.20	9-Methyl heptadecane	0.773ab	0.628ab	0.362b	0.834a
	GC26	17.941	1346.17	N-Hentriacontane	0.395ab	0.107b	0.488a	0.566a
	GC29	18.449	1365.24	9-Methyl heptadecane	0.537a	0.115ab	0.000b	0.382ab
	GC36	19.725	1414.38	Heptadecane	1.155a	0.350ab	0.245b	0.998ab
	GC45	21.22	1470.74	Heptadecane,2,6,10,14-tetramethyl	0.402b	0.665ab	0.961a	0.696ab
	GC47	21.473	1480.44	Tetradecane, 3-methyl	0.520ab	0.245b	0.607a	0.288ab
	GC52	23.034	1544.93	Tetratetracontane	0.000b	0.087ab	0.183a	0.077b
	GC57	23.895	1581.74	Pentadecane, 3-methyl	0.042ab	0.035ab	0.112a	0.000b
	GC61	26.934	1716.38	Hexadecane,2,6,10,14-tetramethyl-	0.000b	0.027a	0.000b	0.000b
	GC64	27.785	1754.78	N-Hentriacontane	0.062a	0.008b	0.000b	0.000b
	GC96	46.06	2715.65	1-Hexacosene	0.000b	0.073b	0.039b	0.252a
	GC99	47.375	–	(E,E,E,E)-Squalene	0.000b	0.199a	0.039ab	0.067ab
**Arenes**	GC25	17.703	1311.97	Naphthalene,1,2,3,4-tetrahydro-1,8-dimethyl	0.000b	0.173a	0.000b	0.000b
**Alcohols**	GC72	34.62	2100.11	1-Heptadecanol	0.221b	0.000b	0.000b	0.695a
	GC78	36.816	2227.94	1-Nonadecanol,1-acetate	1.407a	0.415b	1.584a	1.450a
	GC97	46.489	2753.58	Beta-Sitosterol	0.179b	0.867a	0.834a	0.781a
**Esters**	GC42	20.921	1459.28	Isobutyl tetradecyl carbonate	0.424b	0.634ab	0.920a	0.774ab
	GC67	32.044	1964.22	Dibutyl phthalate	0.000b	0.267a	0.263a	0.154ab
	GC71	34.615	2099.84	13-Tetradecen-1-ol-acetate	0.185b	0.906a	0.686ab	0.335ab
	GC87	40.272	2421.00	2,2′-Methylenebis	0.000b	3.966a	5.507a	4.740a
	GC94	42.962	2539.88	Dioctyl phthalate	0.576ab	0.577ab	0.093b	1.132a
**Acids**	GC76	36.074	2180.31	Stearic acid	0.329b	0.000b	1.499a	0.000b
**Amines**	GC85	39.331	2375.05	Oleamide	0.000b	1.051a	0.000b	0.408ab
**Phenols**	GC92	41.56	2483.20	2,4-bis(1-phenylethyl)phenol	0.000c	0.670a	0.485ab	0.294bc

*ID indicates the identifying number of each soil chemical compound via GC-MS, and peak area (%) represents the mean relative abundance value of three replicates. CK: no *Trichoderma*-inoculation and not mowed; T: *Trichoderma*-inoculation and not mowed; M: mowed and no *Trichoderma*-inoculation; TM: mowed and *Trichoderma*-inoculated. Within a row, values that do not share a letter are significantly different (*P* < 0.05) according to Duncan’s test.*

### Rhizosphere Soil Chemical Compounds and Soil Microbial Community Structure

**FIGURE 2:**
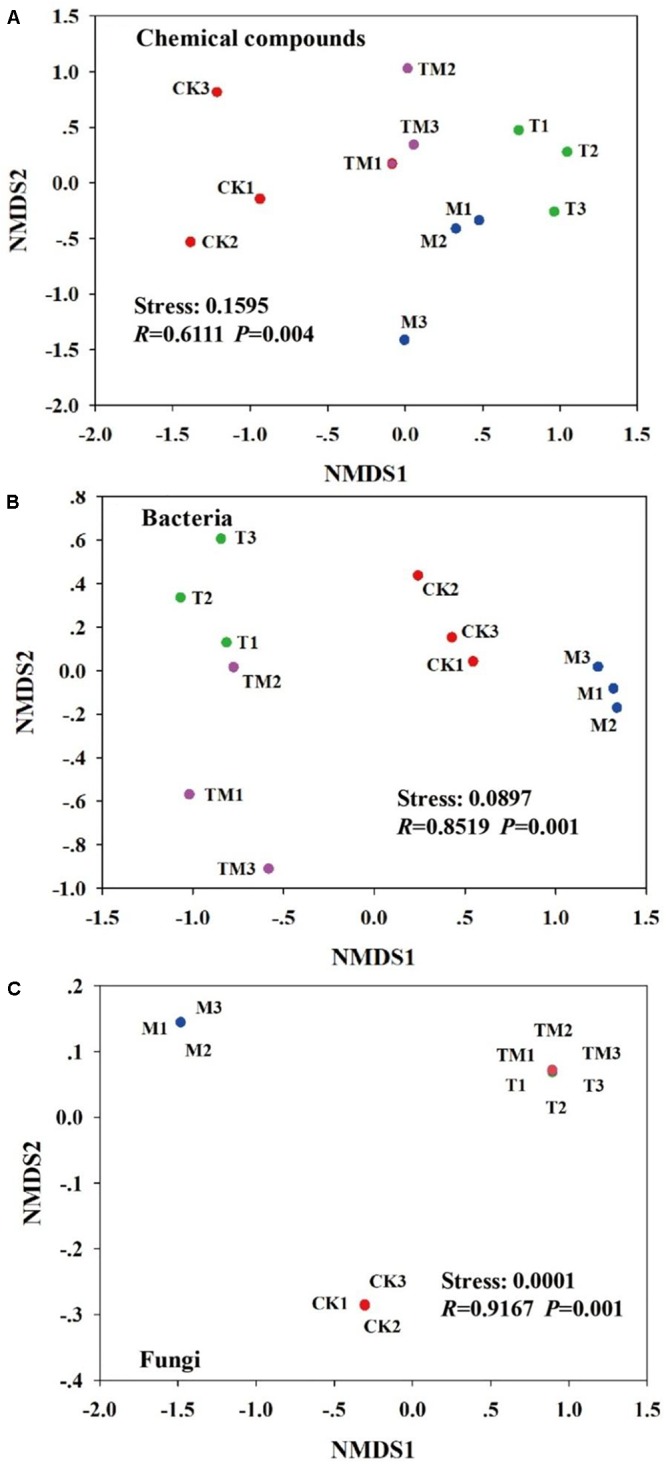
First two non-metric multidimensional scaling (NMDS) axes of the community structure of **(A)** soil chemical compounds, **(B)** bacteria, and **(C)** fungi. Red = CK: no *Trichoderma*-inoculation and not mowed; Green = T: *Trichoderma*-inoculation and not mowed; Blue = M: mowed and no *Trichoderma*-inoculation; Purple = TM: mowed, and *Trichoderma*-inoculated.

As shown in Figure 2, NMDS plots demonstrate the changes in the rhizosphere soil chemical compounds and soil microbial community structure under different treatments. The stress values were all less than 0.2, and the *P*-values were lower than 0.05. The rhizosphere soil chemical compounds were significantly (*P* = 0.004) separated among CK, T, M, and TM plots (Figure 2A). Additionally, the bacterial (*P* = 0.001) and fungal (*P* = 0.001) communities also showed significant differences among the four treatments (Figures 2B,C).

### Bacterial and Fungal Community Composition

**FIGURE 3:**
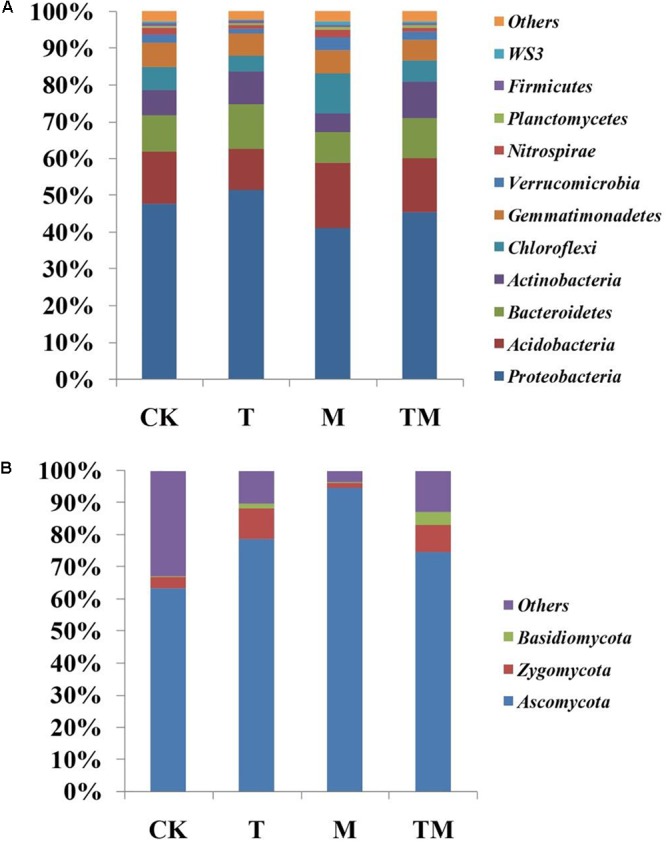
The relative abundance of bacterial phyla **(A)** and fungal phyla **(B)** in different treatments. “Others” indicates phyla with extremely low abundance. CK: no *Trichoderma*-inoculation and not mowed; T: *Trichoderma*-inoculation and not mowed; M: mowed and no *Trichoderma*-inoculation; TM: mowed and *Trichoderma*-inoculated.

The dominant phyla of bacterial and fungal communities in rhizosphere soil showed obvious variations among the four treatments (Figure 3). As shown in Figure 3A, the most abundant bacterial phyla were *Proteobacteria, Acidobacteria, Bacteroidetes, Actinobacteria, Chloroflexi, Gemmatimonadetes, Verrucomicrobia, Nitrospirae, Planctomycetes, Firmicutes*, and *WS3*, which contribute almost 90% of the bacterial sequences. For soil fungal community composition, the fungal sequences were primarily from three phyla: *Ascomycota, Zygomycota*, and *Basidiomycota* (Figure 3B). The relative abundance of *Ascomycota* increased significantly in M treatment (mowed and no *Trichoderma*-inoculation), compared with that in other treatments (CK, T, and TM). At the level of genus, a comparison of the relative abundance of the top 50 classified bacterial and fungal genera revealed significant differences among the four treatments (Supplementary Table S2). We focused on analyzing the soil bacterial and fungal genera that had the ability to potentially promote plants growth. These selected genera showed significant differences among the four treatments (CK, T, M, and TM). As show in Table 3, the inoculation of *Trichoderma* (T) resulted in a significant enrichment of the genera *Pseudomonas, Flavobacterium*, and *Arthrobacter*, while the increasing trend could be weaken when mowing was added (TM). The relative abundance of *Trichoderma* in T and TM treatments were significantly higher than that in CK and M treatments. Moreover, TM treatment had the highest *Trichoderma* relative abundance.

**Table 3:** The relative abundance of selected bacterial and fungal genera of different treatments.

Genera	Treatments				Correlation coefficient	*P*-value
	CK	T	M	TM		
*Pseudomonas*	2.17 ± 0.48b	4.06 ± 0.97a	0.75 ± 0.09c	2.11 ± 0.23b	0.33	0.918
*Flavobacterium*	0.50 ± 0.02b	0.73 ± 0.03a	0.13 ± 0.01c	0.42 ± 0.13b	–0.094	0.771
*Arthrobacter*	0.26 ± 0.13b	0.82 ± 0.49a	0.19 ± 0.06b	0.48 ± 0.09ab	0.301	0.342
*Bacillus*	0.15 ± 0.02a	0.12 ± 0.03ab	0.10 ± 0.01b	0.12 ± 0.02ab	–0.402	0.195
*Trichoderma*	0.01 ± 0.00b	0.10 ± 0.01ab	0.02 ± 0.01b	0.28 ± 0.26a	0.587^∗^	0.045

*CK: no *Trichoderma*-inoculation and not mowed; T: *Trichoderma*-inoculation and not mowed; M: mowed and no *Trichoderma*-inoculation; TM: mowed and *Trichoderma*-inoculated. Data are mean values of three replicates. Numbers followed by “±” are the standard deviations (SDs). Within a row, values that do not share a letter are significantly different (*P* < 0.05). ^∗^Indicates that the difference is statistically significant at the 0.05 probability level, according to Duncan’s test.*

### Coupling the Abundance of Selected Genera and Alfalfa Biomass

**FIGURE 4:**
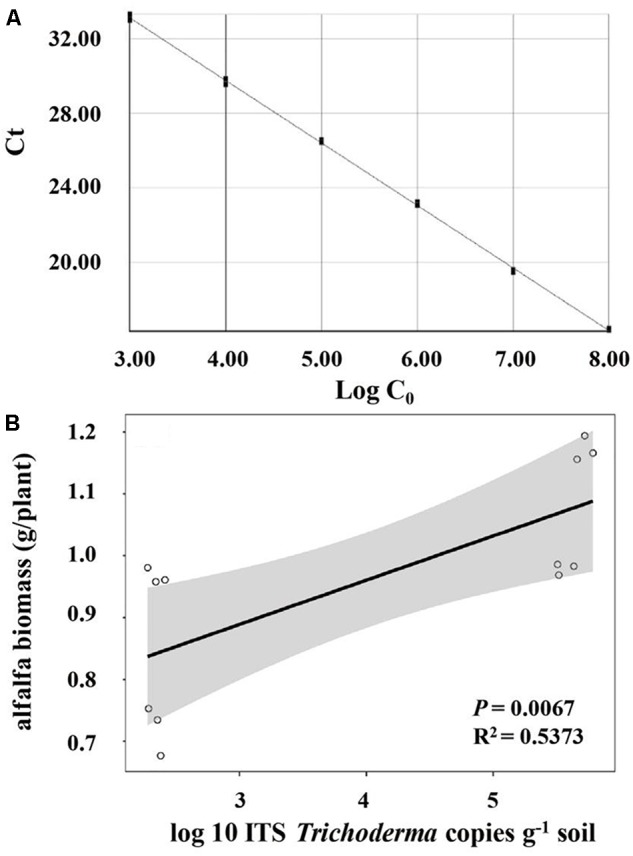
**(A)** Standard curves of *Trichoderma* by real-time PCR; **(B)** correlations between the number of *Trichoderma* and alfalfa biomass.

Significant and positive correlation was observed between the biomass of alfalfa and the relative abundance of *Trichoderma* (*R^2^* = 0.3451, *P* = 0.045) (Table 3). However, *Pseudomonas, Flavobacterium, Arthrobacter, Bacillus, Agrobacterium*, and *Actinoplanes* were not significantly correlated with alfalfa biomass. As shown in the Taq-Man Real-time PCR analysis (Figure 4A), *Trichoderma* had a good linear relationship (*Y* = -3.32X + 43.2, *R^2^* = 0.999) with concentrations of the target gene (*Trichoderma* ITS region). The abundance of *Trichoderma* was also positively (*R^2^* = 0.5373, *P* = 0.0067) correlated with alfalfa biomass (Figure 4B).

### Structure Equation Modeling (SEM) Pathways and Alfalfa Biomass

**FIGURE 5:**
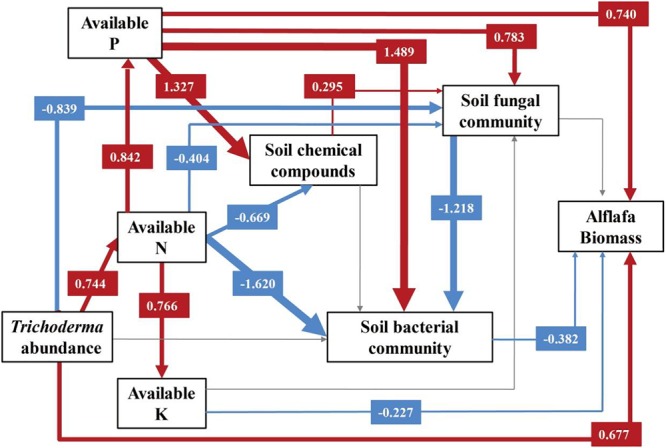
Structural equation modeling (SEM) for alfalfa biomass. A path coefficient is analogous to a partial correlation coefficient and describes the strength and sign of the relationship between two variables. Negative pathways are shown as blue lines, positive pathways are shown as red lines, and line thickness represents the intensity of influence. Non-significant pathways are shown in gray. The models provided a good fit to our data. The model fits are given in Supplementary Table S3, and significance levels are provided in Supplementary Table S4.

Supplementary Table S3 shows the structural equation modeling for alfalfa biomass, which showed a strong fit with the data (*χ*^2^ = 6.336, DF = 8, *P* = 0.610, NFI = 0.967, RFI = 0.886, IFI = 1.009, RMSEA = 0.000, Supplementary Table S4). The variance of alfalfa biomass was directly dependent on *Trichoderma* abundance, available N, P, and K, rhizosphere soil chemical compounds, and soil microbial communities (Figure 5). Among these factors, *Trichoderma* abundance (path coefficient = 0.677) and soil available P (path coefficient = 0.740) had the strongest direct overall effects on alfalfa biomass. However, soil bacterial community (path coefficient = -0.382) and available K (path coefficient = -0.227) had a negative effect on alfalfa biomass. *Trichoderma* abundance had a significant negative effect on soil fungal community (path coefficient = -0.839). Soil chemical compounds was directly influenced by soil available P (path coefficient = 1.327) and available N (path coefficient = -0.669). Soil chemical compounds had a positive influence on soil fungal community (path coefficient = 0.295), while no significant influence was observed on soil bacterial community. The soil bacterial community was directly mediated by available N (path coefficient = -1.620), available P (path coefficient = 1.489) and soil fungal community (path coefficient = -1.218).

## Discussion

In the present study, our results showed that *Trichoderma*-inoculation and/or mowing significantly improved alfalfa growth, with the best results from the alfalfa treated with the *Trichoderma* and mowing treatment (TM). Many reports show that *Trichoderma*-inoculation has significant promotion effects on plants seedlings and crops yields, such as those of cucumber, cotton, tomato, and *Leymus chinensis* (Howell et al., 2000; Bal and Altintas, 2006; Zhang et al., 2013a, 2018a). Contreras-Cornejo et al. (2009) demonstrate that *Trichoderma virens* can stimulate plant lateral root growth by secreting indole acetic acid (IAA). The study of Bjorkman et al. (1998) also shows that *Trichoderma harzianum* has ability to promote root elongation and plants production. *Trichoderma*-inoculation is adept at promoting the growth of plants shoot and root, whereas mowing is commonly beneficial to stimulate aboveground regrowth, which is consistent with our result that the root dry weight in the *Trichoderma*-inoculation treatment (T) was significantly higher than that in mowing treatment (M). Several studies (Yadav et al., 2009; Kapri and Tewari, 2010) demonstrate that *Trichoderma* spp. can increase the solubilization of soil nutrients. Kucuk et al. (2008) also report that the acidification by organic acids released by *T. harzianum* provides more nutrients, directly influencing plants growth. According to systemic induction theory (Scheffknecht et al., 2006; Ling et al., 2011), because mowing directly affects plants aboveground, roots may also be stimulated to secrete more compounds to solubilize nutrients. Such a scenario well explains our result that TM had the greatest contents of available N, P, and K.

The soil environment was complex; thus the rhizosphere soil chemical compounds (e.g., alkanes, arenes, alcohols, esters, phenols and amines) identified by GC-MS were various (99 compounds). *Trichoderma* and/or mowing would lead to plants forming different chemical compounds profile. Many of the identified chemical compounds in CK were less abundant than other treatments, and some chemical compounds (e.g., Tetratetracontane, 1-Hexacosene, and 1-Hexacosene) were not detected in CK, which might be because roots could secrete more compounds when suffering from external environment stress (Liu and Xu, 2014). Compared to other treatments, more abundant 1-Hexacosene and 1-Heptadecanol were detected from TM treatment. Raza et al. (2015) demonstrated that the majority of alcohols have antifungal activity. Thus, we proposed that 1-Heptadecanol might suppress pathogen invasion to indirectly protect alfalfa growth. However, there was little evidence to demonstrate the effect of 1-Hexacosene on plants growth. We should further explore the function of different compounds on plants growth in our future research. In this study, both considerable differentiation of the rhizosphere soil chemical compounds composition and microbial structure were observed among the four treatments (CK, M, T, and MT). Thus, the rhizosphere of each treatment formed a specific and distinct resources niche. The compounds released into the rhizosphere by plant roots are crucial in structuring the soil microbiome composition (Lagos et al., 2014; Carvalhais et al., 2015). The application of artificial root secretions to soil samples can also considerably mediate the soil microbiome composition (Paterson et al., 2007; Eilers et al., 2010). A number of studies (Bernard et al., 2007; Paterson et al., 2007) demonstrate that the addition of low molecular weight organic carbon compounds to soil can directly and indirectly increase microbial biomass and cause distinct changes in the composition of bacterial communities. Gu et al. (2016) also show that changes in the root exudation profile shift the composition of rhizosphere bacterial communities. These results are consistent with our study that a corresponding differentiation of bacterial and fungal community compositions are based on rhizosphere chemical compounds differences among treatments. Note that mowing itself can change the secondary metabolism of plants, and also has influence on some plant physiology process (i.e., carbon assimilation) to change the composition of microbes which produced different compounds. Thus, some detected chemical compounds may be of microbial origin in our study. Future experiments should be conducted to clarify the exact origin of the rhizosphere chemical compounds.

Significant differences were observed in soil bacterial and fungal phyla and genera compositions among the four treatments. The dominant bacterial phyla were *Proteobacteria, Acidobacteria, Bacteroidetes*, and *Actinobacteria*, which was consistent with previous studies (Janssen, 2006; Lauber et al., 2009). Among these, *Proteobacteria* was the most dominant bacterial phylum across all samples, which was most likely due to its relatively rapid growth rate (Fierer et al., 2007; DeAngelis et al., 2008). As for the fungal phyla, *Ascomycota* was the most abundant in the rhizosphere based on deep pyrosequencing analysis of fungal community composition, which is consistent with the results of many reports (Yu et al., 2013; Shen et al., 2015). The inoculated strain *T. harzianum* belongs to *Ascomycota* phylum, which mainly account for that the relative abundance of *Ascomycota* in T was significantly higher than CK. *Trichoderma spp.* also have been reported as bio-agents and show potent antagonistic effect against a variety of soil pathogens (Howell et al., 2000; Zhang et al., 2013b). According to our results that the relative abundance of *Ascomycota* in M was significantly higher than TM, we concluded that mowing might have the ability to increase the relative abundance of soil pathogens, and *Trichoderma* play an antagonistic role in TM. The soil bacterial community compositions vary in different treatments, where the antagonism may take place between the inoculated-*Trichoderma* and some bacteria (Pan and Jash, 2011). We further focused on determining the variations of some microbial genera (*Pseudomonas, Flavobacterium, Arthrobacter, Bacillus, Agrobacterium, Actinoplanes*, and *Trichoderma*) that potentially promote plants growth. Alfalfa biomass had significant correlation with the relative abundance of *Trichoderma*. However, the relative abundances which are acquired from the high-throughput sequencing are not suitable to quantify when using universal primers, and the relative read abundance of individual species is influenced by other species (Amend et al., 2010). Thus, we further employed Real-time PCR to quantify *Trichoderma* in rhizosphere soil. The quantitative determination showed that alfalfa biomass was positively correlated with the abundance of *Trichoderma*, which was consistent with our previous relative abundance result.

The SEM results showed that *Trichoderma* abundance, available N, P, and K, soil chemical compounds, and fungal and bacterial community explain for alfalfa biomass in the pot experiment. *Trichoderma* greatly influenced alfalfa biomass, which was primarily due to its characteristic of plant growth promotion in greenhouse or field conditions. The soil available P also had a positive effect on alfalfa biomass, and an increase in available P was directly beneficial to plant growth. This supports our results that the soil available P content was significantly higher in the TM treatment than that in other treatments and that TM had the highest alfalfa biomass. The soil fungal community was most closely associated with changes in soil nutrients (Christian et al., 2008). The inoculation of *Trichoderma* had a negative influence on the soil fungal community, which was consistent with our result in a previous study (Zhang et al., 2018a). Raaijmakers et al. (2009) revealed that plant root exudates, as the major C source of soil microbes could directly influence their assemblage. According to SEM, we concluded that soil chemical compounds have direct effect on soil fungal community, while indirect influence on soil bacterial community.

*Trichoderma*-inoculation and mowing synergistically regulated soil available nutrients, rhizosphere soil chemical compounds and soil microbial community, substantially improved alfalfa growth, with *Trichoderma* abundance and soil available P as the primary contributors. Additionally, *Trichoderma*-inoculation and mowing altered rhizosphere soil chemical compounds to influence the soil microbial community, indirectly influencing alfalfa growth. Our research provides a basis for promoting alfalfa growth from the perspective of soil microbial ecology and may ultimately enrich promotion theory. Furthermore, studies should be conducted in the future to improve understanding of the functional consequences of *Trichoderma*-inoculation and/or mowing amendments on the soil microbial community and how these effects result in alteration of soil properties to support plant growth.

## Author Contributions

FZ, YH, and YX conceived and designed the experiments. FZ, XX, and YH performed the experiments. FZ and XX analyzed the data. FZ and YX contributed reagents, materials, and analysis tools and wrote the paper. All authors reviewed and contributed to the manuscript.

## Conflict of Interest Statement

The authors declare that the research was conducted in the absence of any commercial or financial relationships that could be construed as a potential conflict of interest.
