# Supplementary material for: Trichoderma-Inoculation and Mowing Synergistically Altered Soil Available Nutrients, Rhizosphere Chemical Compounds and Soil Microbial Community, Potentially Driving Alfalfa Growth
Source: Front Microbiol. 2019 Jan 7;9:3241. doi: 10.3389/fmicb.2018.03241 (PMC6330351; doi:10.3389/fmicb.2018.03241)
Supplement: Supplementary file 1 [file Data_Sheet_1.docx]

**Supporting information**

**Fig. S1** Phylogenetic tree for partial sequences of cloned ITS regions and most closely related fungi. Clones are indicated by their code and accession number (NCBI)


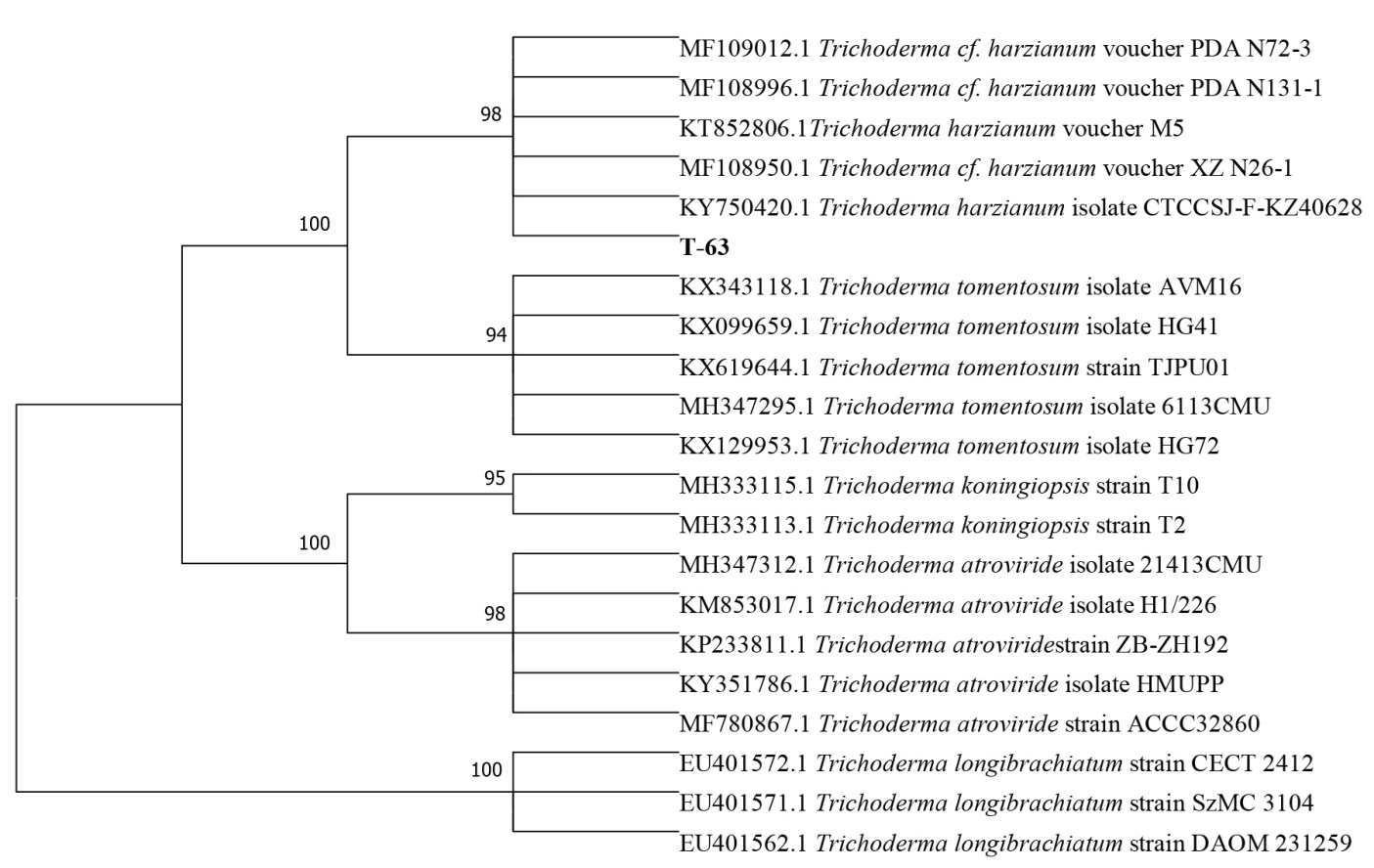


**Fig. S1**

**Table S1** Results of GC-MS identification of soil chemicals according to the National Institute of Standards and Technology database

| **ID** | **Retention time** | **Peak area (%)** | | | |
| --- | --- | --- | --- | --- | --- |
|  |  | **CK** | **T** | **M** | **TM** |
| GC1 | 10.415 | 0.123 | 0.057 | 0.080 | 0.067 |
| GC2 | 10.590 | 0.000 | 0.092 | 0.000 | 0.082 |
| GC3 | 10.972 | 0.000 | 0.014 | 0.003 | 0.011 |
| GC4 | 12.069 | 0.159 | 0.110 | 0.005 | 0.196 |
| GC5 | 12.171 | 0.098 | 0.256 | 0.228 | 0.287 |
| GC6 | 12.308 | 0.092 | 0.057 | 0.147 | 0.262 |
| GC7 | 12.722 | 0.183 | 0.106 | 0.194 | 0.000 |
| GC8 | 12.872 | 0.577 | 0.499 | 0.337 | 0.687 |
| GC9 | 13.343 | 0.711 | 0.659 | 0.110 | 0.588 |
| GC10 | 14.216 | 0.509 | 0.530 | 0.470 | 0.611 |
| GC11 | 14.593 | 1.021 | 0.422 | 0.143 | 1.11 |
| GC12 | 14.795 | 0.329 | 0.000 | 0.277 | 0.000 |
| GC13 | 14.997 | 0.040 | 0.235 | 0.370 | 0.000 |
| GC14 | 15.337 | 0.558 | 0.443 | 0.540 | 0.445 |
| GC15 | 15.675 | 0.345 | 0.404 | 0.038 | 0.467 |
| GC16 | 15.738 | 0.304 | 0.343 | 0.576 | 0.000 |
| GC17 | 15.88 | 0.773 | 0.628 | 0.362 | 0.834 |
| GC18 | 16.259 | 1.489 | 1.304 | 1.474 | 1.508 |
| GC19 | 16.363 | 0.256 | 0.447 | 0.293 | 0.837 |
| GC20 | 16.717 | 0.399 | 0.159 | 0.000 | 0.000 |
| GC21 | 17.029 | 0.810 | 0.789 | 0.891 | 0.839 |
| GC22 | 17.475 | 0.505 | 0.000 | 0.430 | 0.000 |
| GC23 | 17.489 | 0.402 | 0.598 | 0.691 | 0.793 |
| GC24 | 17.536 | 0.117 | 0.076 | 0.000 | 0.203 |
| GC25 | 17.703 | 0.000 | 0.173 | 0.000 | 0.000 |
| GC26 | 17.941 | 0.395 | 0.107 | 0.488 | 0.566 |
| GC27 | 18.209 | 0.554 | 0.634 | 0.619 | 0.730 |
| GC28 | 18.345 | 0.657 | 0.594 | 0.813 | 0.621 |
| GC29 | 18.449 | 0.537 | 0.115 | 0.000 | 0.382 |
| GC30 | 18.588 | 0.864 | 0.654 | 0.318 | 0.551 |
| GC31 | 18.75 | 1.112 | 0.602 | 1.108 | 0.938 |
| GC32 | 18.919 | 0.770 | 0.618 | 0.831 | 0.717 |
| GC33 | 19.058 | 1.393 | 1.111 | 1.556 | 1.215 |
| GC34 | 19.299 | 0.300 | 0.366 | 0.475 | 0.333 |
| GC35 | 19.465 | 0.186 | 0.194 | 0.000 | 0.000 |
| GC36 | 19.725 | 1.155 | 0.350 | 0.245 | 0.998 |
| GC37 | 19.92 | 0.093 | 0.000 | 0.409 | 0.208 |
| GC38 | 19.7 | 0.000 | 0.183 | 0.000 | 0.000 |
| GC39 | 20.037 | 0.175 | 0.415 | 0.000 | 0.290 |
| GC40 | 20.356 | 0.389 | 0.395 | 0.567 | 0.341 |
| GC41 | 20.611 | 0.226 | 0.000 | 0.118 | 0.134 |
| GC42 | 20.921 | 0.424 | 0.634 | 0.920 | 0.774 |
| GC43 | 21.048 | 0.357 | 0.052 | 0.008 | 0.273 |
| GC44 | 21.152 | 0.212 | 0.162 | 0.160 | 0.337 |
| GC45 | 21.22 | 0.402 | 0.665 | 0.961 | 0.696 |
| GC46 | 21.31 | 0.637 | 0.500 | 0.690 | 0.592 |
| GC47 | 21.473 | 0.520 | 0.245 | 0.607 | 0.288 |
| GC48 | 21.695 | 0.109 | 0.152 | 0.185 | 0.042 |
| GC49 | 21.905 | 0.160 | 0.170 | 0.315 | 0.207 |
| GC50 | 22.061 | 0.000 | 0.124 | 0.000 | 0.077 |
| GC51 | 22.216 | 0.690 | 0.397 | 0.770 | 0.594 |
| GC52 | 23.034 | 0.000 | 0.087 | 0.183 | 0.077 |
| GC53 | 23.322 | 0.112 | 0.131 | 0.133 | 0.128 |
| GC54 | 23.466 | 0.209 | 0.170 | 0.148 | 0.171 |
| GC55 | 23.621 | 0.078 | 0.052 | 0.009 | 0.063 |
| GC56 | 23.754 | 0.127 | 0.135 | 0.106 | 0.080 |
| GC57 | 23.895 | 0.042 | 0.035 | 0.112 | 0.000 |
| GC58 | 24.574 | 0.135 | 0.143 | 0.089 | 0.137 |
| GC59 | 25.664 | 0.021 | 0.026 | 0.024 | 0.055 |
| GC60 | 26.855 | 0.068 | 0.078 | 0.106 | 0.046 |
| GC61 | 26.934 | 0.000 | 0.027 | 0.000 | 0.000 |
| GC62 | 27.145 | 0.024 | 0.027 | 0.045 | 0.027 |
| GC63 | 27.355 | 0.117 | 0.219 | 0.238 | 0.122 |
| GC64 | 27.785 | 0.062 | 0.008 | 0.000 | 0.000 |
| GC65 | 29.827 | 0.036 | 0.000 | 0.029 | 0.000 |
| GC66 | 30.139 | 0.040 | 0.065 | 0.007 | 0.080 |
| GC67 | 32.044 | 0.000 | 0.267 | 0.263 | 0.154 |
| GC68 | 32.288 | 0.197 | 0.403 | 0.270 | 0.784 |
| GC69 | 32.804 | 0.043 | 0.272 | 0.080 | 0.199 |
| GC70 | 33.675 | 0.252 | 0.455 | 0.381 | 0.181 |
| GC71 | 34.615 | 0.185 | 0.906 | 0.686 | 0.335 |
| GC72 | 34.62 | 0.221 | 0.000 | 0.000 | 0.695 |
| GC73 | 35.033 | 0.530 | 0.317 | 0.000 | 0.000 |
| GC74 | 35.262 | 0.292 | 0.923 | 0.805 | 0.600 |
| GC75 | 35.599 | 0.208 | 0.191 | 0.088 | 0.176 |
| GC76 | 36.074 | 0.329 | 0.000 | 1.499 | 0.000 |
| GC77 | 36.246 | 0.771 | 1.214 | 0.000 | 0.988 |
| GC78 | 36.816 | 1.407 | 0.415 | 1.584 | 1.450 |
| GC79 | 36.99 | 0.557 | 0.545 | 0.783 | 0.274 |
| GC80 | 37.301 | 1.028 | 0.395 | 0.705 | 0.390 |
| GC81 | 37.325 | 0.205 | 0.529 | 0.000 | 0.643 |
| GC82 | 37.536 | 0.787 | 0.000 | 0.621 | 0.419 |
| GC83 | 37.548 | 0.000 | 0.847 | 0.572 | 0.402 |
| GC84 | 38.423 | 0.366 | 0.749 | 0.424 | 0.230 |
| GC85 | 39.331 | 0.000 | 1.051 | 0.000 | 0.408 |
| GC86 | 39.884 | 0.172 | 0.473 | 0.000 | 0.130 |
| GC87 | 40.272 | 0.000 | 3.966 | 5.507 | 4.740 |
| GC88 | 40.948 | 0.000 | 0.354 | 0.268 | 0.000 |
| GC89 | 40.974 | 0.000 | 0.217 | 0.000 | 0.274 |
| GC90 | 41.249 | 0.000 | 0.296 | 0.083 | 1.343 |
| GC91 | 41.282 | 0.477 | 0.340 | 0.647 | 0.652 |
| GC92 | 41.56 | 0.000 | 0.670 | 0.485 | 0.294 |
| GC93 | 42.150 | 0.084 | 0.243 | 0.185 | 0.181 |
| GC94 | 42.962 | 0.576 | 0.577 | 0.093 | 1.132 |
| GC95 | 45.771 | 0.000 | 0.156 | 0.039 | 0.275 |
| GC96 | 46.06 | 0.000 | 0.073 | 0.039 | 0.252 |
| GC97 | 46.489 | 0.179 | 0.867 | 0.834 | 0.781 |
| GC98 | 47.071 | 0.362 | 0.123 | 0.000 | 0.250 |
| GC99 | 47.375 | 0.000 | 0.199 | 0.039 | 0.067 |

Notes: ID indicates the identifying number of each soil chemical via GC-MS, and peak area represents the mean relative abundance value of three replicates. CK: no *Trichoderma*-inoculation and not mowed; T: *Trichoderma*-inoculation and not mowed; M: mowed and no *Trichoderma*-inoculation; TM: mowed and *Trichoderma*-inoculated. Data are mean values of three replicates.

**Table S2** The relative abundance (%) of the top 50 classified bacterial and fungal genera of different treatments

| **Bacteria** | | | | | **Fungi** | | | | |
| --- | --- | --- | --- | --- | --- | --- | --- | --- | --- |
| **Genera** | **CK** | **T** | **M** | **TM** | **Genera** | **CK** | **T** | **M** | **TM** |
| *Pseudomonas* | 2.17±0.28b | 4.06±0.56a | 0.75±0.05c | 2.11±0.13b | *Fusarium* | 4.22±1.48a | 3.56±0.57ab | 1.12±0.28b | 5.08±0.39a |
| *Kaistobacter* | 1.39±0.07c | 2.24±0.11a | 1.03±0.02b | 1.68±0.10d | *Mortierella* | 1.95±0.04b | 5.96±0.75a | 0.69±0.02b | 4.90±0.43a |
| *Lysobacter* | 1.08±0.07bc | 1.49±0.10a | 0.95±0.04c | 1.20±0.07b | *Alternaria* | 8.53±2.18a | 0.33±0.09b | 0.86±0.03b | 0.99±0.51b |
| *Rhodoplanes* | 1.01±0.05a | 0.84±0.02b | 0.87±0.03b | 0.71±0.03c | *Neonectria* | 0.64±0.32b | 5.72±1.47a | 0.10±0.09b | 2.10±1.05b |
| *Thermomonas* | 0.47±0.03b | 1.13±0.01a | 0.53±0.02b | 1.06±0.11a | *Myrothecium* | 3.30±1.27a | 3.72±2.76a | 1.02±0.53a | 0.41±0.03a |
| *Flavisolibacter* | 0.50±0.01b | 0.73±0.02a | 0.13±0.00b | 0.42±0.07a | *Mortierella* | 1.39±0.11b | 3.06±0.60a | 0.80±0.04b | 2.71±0.16a |
| *Nitrospira* | 0.65±0.02b | 0.39±0.02c | 0.82±0.02a | 0.32±0.03c | *Pyrenochaeta* | 0.15±0.13a | 6.81±5.94a | 0.31±0.17a | 0.25±0.11a |
| *Sphingomonas* | 0.38±0.02c | 0.77±0.04a | 0.29±0.03d | 0.67±0.02b | *Plectosphaerella* | 1.74±0.13a | 0.53±0.07b | 1.15±0.17ab | 1.02±0.43ab |
| *Janthinobacterium* | 0.47±0.05b | 0.64±0.06a | 0.29±0.01c | 0.62±0.03a | *Volutella* | 2.32±0.24a | 0.51±0.05bc | 0.23±0.12c | 0.91±0.22b |
| *Hyphomicrobium* | 0.52±0.02a | 0.48±0.01ab | 0.43±0.02bc | 0.40±0.04c | *Penicillium* | 0.11±0.01b | 1.22±0.30a | 0.13±0.01b | 1.59±0.42a |
| *Streptomyces* | 0.34±0.02c | 0.76±0.04a | 0.21±0.02d | 0.52±0.06b | *Pseudogymnoascus* | 2.10±1.09a | 0.25±0.03b | 0.02±0.00b | 0.15±0.07b |
| *Flavobacterium* | 0.50±0.01b | 0.73±0.02a | 0.13±0.00c | 0.42±0.07b | *Clonostachys* | 0.10±0.02b | 0.82±0.29ab | 0.06±0.02b | 1.10±0.73a |
| *Arthrobacter* | 0.26±0.07b | 0.82±0.28a | 0.19±0.04b | 0.48±0.05ab | *Cladosporium* | 1.07±0.16a | 0.26±0.07b | 0.48±0.07b | 0.24±0.01b |
| *Novosphingobium* | 0.42±0.04b | 0.58±0.05a | 0.29±0.02c | 0.43±0.01b | *Staphylotrichum* | 0.08±0.01c | 0.56±0.21b | 0.08±0.01c | 1.20±0.16a |
| *Candidatus Nitrososphaera* | 0.59±0.34a | 0.31±0.19a | 0.44±0.08a | 0.37±0.11a | *Gibberella* | 0.37±0.04bc | 0.48±0.08ab | 0.22±0.01c | 0.64±0.08a |
| *Steroidobacter* | 0.48±0.01a | 0.41±0.04ab | 0.46±0.03ab | 0.36±0.02b | *Preussia* | 0.34±0.16a | 0.57±0.30a | 0.14±0.02a | 0.41±0.09a |
| *Bradyrhizobium* | 0.46±0.03a | 0.46±0.03a | 0.36±0.01b | 0.39±0.01ab | *Madurella* | 0.08±0.01bc | 0.49±0.10ab | 0.05±0.01c | 0.81±0.23a |
| *Rhodobacter* | 0.22±0.01b | 0.66±0.04a | 0.19±0.01b | 0.55±0.06a | *Lulworthia* | 0.04±0.03b | 0.38±0.14ab | 0.02±0.02b | 0.93±0.48a |
| *Candidatus Solibacter* | 0.42±0.03b | 0.27±0.03c | 0.51±0.03a | 0.28±0.01c | *Tetracladium* | 0.08±0.01a | 0.60±0.32a | 0.11±0.00a | 0.44±0.23a |
| *Mycoplana* | 0.33±0.02b | 0.58±0.02a | 0.12±0.00c | 0.41±0.04b | *Villosiclava* | 0.02±0.01b | 0.27±0.04b | 0.02±0.00b | 0.85±0.33a |
| *Sphingopyxis* | 0.33±0.04b | 0.54±0.06a | 0.07±0.01c | 0.33±0.01b | *Podospora* | 0.09±0.03b | 0.47±0.09a | 0.05±0.02b | 0.56±0.11a |
| *Rubrivivax* | 0.29±0.03ab | 0.23±0.03b | 0.31±0.02a | 0.22±0.00b | *Didymosphaeria* | 0.02±0.00b | 0.44±0.13a | 0.05±0.00b | 0.56±0.07a |
| *Phenylobacterium* | 0.16±0.02bc | 0.29±0.05a | 0.16±0.00c | 0.25±0.03ab | *Westerdykella* | 0.02±0.00c | 0.47±0.06a | 0.04±0.01c | 0.32±0.06b |
| *Skermanella* | 0.17±0.02b | 0.28±0.01a | 0.12±0.01b | 0.28±0.04a | *Ilyonectria* | 0.10±0.00b | 0.42±0.09a | 0.06±0.00b | 0.28±0.02a |
| *Arenimonas* | 0.13±0.01b | 0.28±0.03a | 0.11±0.01b | 0.29±0.04a | *Mortierella* | 0.09±0.03c | 0.38±0.06a | 0.04±0.01c | 0.26±0.03b |
| *Pseudoxanthomonas* | 0.25±0.02b | 0.32±0.03a | 0.05±0.01c | 0.20±0.02b | *Mortierella* | 0.11±0.01c | 0.28±0.05b | 0.04±0.01c | 0.29±0.03a |
| *GOUTA19* | 0.29±0.03a | 0.10±0.01b | 0.32±0.02a | 0.10±0.01b | *Arthrographis* | 0.65±0.31a | 0.02±0.01b | 0.00±0.00b | 0.03±0.01b |
| *Thiobacillus* | 0.26±0.01a | 0.11±0.02b | 0.28±0.03a | 0.15±0.02b | *Scleroderma* | 0.01±0.00a | 0.24±0.15a | 0.04±0.01a | 0.30±0.23a |
| *Anaeromyxobacter* | 0.23±0.01a | 0.15±0.02b | 0.26±0.01a | 0.15±0.03b | *Acremonium* | 0.41±0.28a | 0.04±0.01a | 0.02±0.00a | 0.12±0.04a |
| *Geobacter* | 0.25±0.01a | 0.14±0.01b | 0.25±0.00a | 0.13±0.01b | *Talaromyces* | 0.03±0.00b | 0.18±0.05ab | 0.01±0.00b | 0.36±0.14a |
| *Agromyces* | 0.14±0.01b | 0.24±0.02a | 0.08±0.01c | 0.25±0.02a | *Lecythophora* | 0.02±0.00a | 0.18±0.07a | 0.03±0.02a | 0.35±0.19a |
| *Sphingobium* | 0.11±0.02a | 0.10±0.02a | 0.07±0.01a | 0.42±0.34a | *Hypocrea* | 0.03±0.00b | 0.25±0.02a | 0.03±0.00b | 0.24±0.03a |
| *Stenotrophomonas* | 0.21±0.02a | 0.23±0.04a | 0.03±0.01b | 0.19±0.04a | *Zopfiella* | 0.04±0.01a | 0.17±0.09a | 0.15±0.01a | 0.17±0.07a |
| *Adhaeribacter* | 0.14±0.00a | 0.17±0.01a | 0.15±0.01a | 0.17±0.01a | *Metarhizium* | 0.04±0.03b | 0.25±0.09a | 0.05±0.04ab | 0.16±0.06ab |
| *Flavihumibacter* | 0.10±0.02b | 0.24±0.03a | 0.09±0.01b | 0.18±0.02a | *Stachybotrys* | 0.06±0.02a | 0.14±0.07a | 0.15±0.03a | 0.11±0.03a |
| *Parasegitibacter* | 0.07±0.01b | 0.22±0.02a | 0.08±0.01b | 0.21±0.01a | *Trichoderma* | 0.01±0.00b | 0.10±0.00ab | 0.02±0.01b | 0.28±0.15a |
| *Pedobacter* | 0.12±0.01b | 0.27±0.01a | 0.02±0.00c | 0.15±0.03b | *Cercophora* | 0.01±0.01a | 0.02±0.00a | 0.01±0.01a | 0.34±0.31a |
| *Euzebya* | 0.14±0.01b | 0.13±0.01b | 0.03±0.00c | 0.24±0.02a | *Bionectria* | 0.02±0.00c | 0.08±0.03b | 0.01±0.00c | 0.28±0.02a |
| *Rhodocytophaga* | 0.07±0.02b | 0.20±0.04a | 0.04±0.00b | 0.21±0.07a | *Scutellinia* | 0.00±0.00a | 0.09±0.02a | 0.00±0.00a | 0.27±0.17a |
| *Anaerolinea* | 0.12±0.04ab | 0.07±0.02b | 0.21±0.04a | 0.10±0.03b | *Aspergillus* | 0.01±0.00a | 0.19±0.11a | 0.01±0.00a | 0.13±0.04a |
| *Pontibacter* | 0.07±0.00ab | 0.15±0.01c | 0.09±0.03bc | 0.18±0.01a | *Pseudeurotium* | 0.02±0.00b | 0.12±0.04a | 0.01±0.01b | 0.18±0.04a |
| *Nannocystis* | 0.09±0.02bc | 0.19±0.02a | 0.07±0.02c | 0.14±0.03ab | *Hymenochaete* | 0.02±0.01b | 0.08±0.05b | 0.01±0.00b | 0.21±0.01a |
| *Bacillus* | 0.15±0.01a | 0.12±0.02ab | 0.10±0.01b | 0.12±0.01ab | *Chaetomium* | 0.01±0.00a | 0.15±0.05a | 0.01±0.00a | 0.15±0.08a |
| *Sporosarcina* | 0.12±0.01a | 0.11±0.02a | 0.10±0.01a | 0.14±0.02a | *Stachybotrys* | 0.02±0.01b | 0.11±0.03a | 0.01±0.00b | 0.09±0.02a |
| *Mycobacterium* | 0.11±0.01bc | 0.14±0.00a | 0.09±0.00c | 0.12±0.01ab | *Emericella* | 0.00±0.00b | 0.15±0.05a | 0.01±0.00b | 0.08±0.03ab |
| *Balneimonas* | 0.09±0.00b | 0.16±0.01a | 0.06±0.01b | 0.14±0.02a | *Pseudallescheria* | 0.00±0.00a | 0.05±0.05a | 0.00±0.00a | 0.16±0.14a |
| *Polaromonas* | 0.11±0.01b | 0.14±0.01a | 0.10±0.01b | 0.09±0.01b | *Monoblepharella* | 0.00±0.00a | 0.08±0.06a | 0.00±0.00a | 0.13±0.05a |
| *Afifella* | 0.13±0.00a | 0.12±0.00a | 0.09±0.00b | 0.09±0.01b | *Coprinopsis* | 0.00±0.00c | 0.06±0.02ab | 0.01±0.00bc | 0.13±0.07a |
| *Methylotenera* | 0.11±0.01ab | 0.13±0.01a | 0.09±0.01b | 0.10±0.01ab | *Massarina* | 0.00±0.00b | 0.05±0.01ab | 0.01±0.00b | 0.12±0.05a |
| *Phormidium* | 0.01±0.00a | 0.11±0.03a | 0.04±0.02a | 0.25±0.16a | *Coprinellus* | 0.00±0.00a | 0.14±0.14a | 0.01±0.01a | 0.02±0.01a |

Notes: CK: no *Trichoderma*-inoculation and not mowed; T: *Trichoderma*-inoculation and not mowed; M: mowed and no *Trichoderma*-inoculation; TM: mowed and *Trichoderma*-inoculated. Data are mean values of three replicates. Numbers followed by “±” are the standard errors (SEs).Within a row, values that do not share a letter are significantly different (*P* < 0.05) according to Duncan’s test.

**Table S3** Indices of fit for structural equation modelling provided in **Fig. 5**.

| **Fit index** | χ^2^ | ***P*** | **DF** | **NFI** | **RFI** | **IFI** | **RMSEA** | **AIC** | **ECVI** |
| --- | --- | --- | --- | --- | --- | --- | --- | --- | --- |
| Result | 6.336 | 0.610 | 8 | 0.967 | 0.886 | 1.009 | 0.000 | 78.336 | 7.121 |

Note: χ^2^ = chi-square (minimum function test statistic); DF = degrees of freedom; NFI = normed fit index; RFI = relative fit index; IFI = incremental index of fit; RMSEA = root mean square error of approximation; AIC = Akaike information criteria; ECVI = expected cross-validation index.

**Table S4** Significance levels for the influences of biotic and abiotic factors on alfalfa biomass, based on structural equation modelling (SEM)

|  |  |  | **Estimate** | **S.E.** | **C.R.** | **P** |
| --- | --- | --- | --- | --- | --- | --- |
| Z Available N | <--- | Z *Trichoderma* abundance | .744 | .201 | 3.697 | *** |
| Z Available P | <--- | Z Available N | .842 | .163 | 5.176 | *** |
| Z Available K | <--- | Z Available N | .766 | .194 | 3.949 | *** |
| Z Soil chemicals community | <--- | Z Available N | -.669 | .299 | -2.239 | .025 |
| Z Soil chemicals community | <--- | Z Available P | 1.327 | .299 | 4.441 | *** |
| Z Soil fungal community | <--- | Z *Trichoderma* abundance | -.839 | .079 | -10.603 | *** |
| Z Soil fungal community | <--- | Z Available P | .783 | .161 | 4.849 | *** |
| Z Soil fungal community | <--- | Z Soil chemicals community | .295 | .096 | 3.077 | .002 |
| Z Soil fungal community | <--- | Z Available K | -.118 | .077 | -1.523 | .128 |
| Z Soil fungal community | <--- | Z Available N | -.404 | .150 | -2.700 | .007 |
| Z Soil bacterial community | <--- | Z *Trichoderma* abundance | .384 | .199 | 1.935 | .053 |
| Z Soil bacterial community | <--- | Z Available N | -1.620 | .154 | -10.520 | *** |
| Z Soil bacterial community | <--- | Z Available P | 1.489 | .211 | 7.068 | *** |
| Z Soil bacterial community | <--- | Z Soil fungal community | -1.218 | .219 | -5.561 | *** |
| Z Soil bacterial community | <--- | Z Soil chemiscals community | -.154 | .103 | -1.493 | .135 |
| Z alfalfa biomass | <--- | Z *Trichoderma* abundance | .677 | .092 | 7.337 | *** |
| Z alfalfa biomass | <--- | Z Available P | .740 | .073 | 10.085 | *** |
| Z alfalfa biomass | <--- | Z Soil bacterial community | -.382 | .052 | -7.282 | *** |
| Z alfalfa biomass | <--- | Z Soil fungal community | -.133 | .070 | -1.911 | .056 |
| Z alfalfa biomass | <--- | Z Available K | -.227 | .040 | -5.704 | *** |

Notes: Significance. codes: ***0.001, *p*-values based on 999 permutations**.**
